# Moral Judgments Depend on Information Presentation: Evidence for Recency and Transfer Effects

**DOI:** 10.5334/pb.421

**Published:** 2018-09-27

**Authors:** Laëtitia Leloup, Gaëlle Meert, Dana Samson

**Affiliations:** 1 Psychological Sciences Research Institute, Université catholique de Louvain, Louvain-la-Neuve, BE; 2 Institute of Neuroscience, Université catholique de Louvain, Bruxelles, BE

**Keywords:** moral judgement, within-scenario order effect, between-scenarios order effect, recency effect, transfer effect

## Abstract

Moral judgements are crucial for social life and rely on the analysis of the agent’s intention and the outcome of the agent’s action. The current study examines to the influence of how the information is presented on moral judgement. The first experiment investigated the effects of the order in which intention and outcome information was presented. The results showed that participants relied more on the last presented information, suggesting a recency effect. The second experiment required participants to make two types of judgments (wrongness vs. punishment) and manipulated the order of the requested two types of judgments. Results showed an asymmetrical transfer effect whereby punishment judgements, but not wrongness judgements were affected by the order of presentation. This asymmetrical transfer effect was likely linked to the ambiguity of the punishment judgement. Altogether, the study showed that the order in which information was presented and the order in which one was asked to think about the wrongness of an action or the punishment that the action deserves were two factors that should be irrelevant, but actually influenced moral judgements. The influence of these factors was mostly observed during the most difficult judgements, precisely in situations where human decision is called upon, such as in court trials.

## Introduction

Moral judgements play an important role in our daily life. They allow us to detect individuals who are responsible for harmful or potentially harmful actions, to hold them accountable for their actions and to punish them. At the collective level, sanctions such as imprisonment, fines or blame discourage harmful behaviors and maintain social order. At the individual level, the identification of moral transgressors who threaten our well-being allows us to avoid or ostracize these transgressors (Alicke, 2000).

The current study aimed at examining the extent to which moral judgements are based solely on relevant principles. Imagine that as a jury member, you have to decide the sentence for a man who was accused of having accidentally hurt his girlfriend. You know that the man had no intention to hurt his girlfriend (intention) and you know that the girl was badly hurt (outcome). How much punishment do you think this man deserves? To what extent will factors unrelated to the protagonists or the circumstances of the action have an impact on your judgement? Here, the study manipulated two factors that should be irrelevant to moral judgements: the order in which intention and outcome information was presented and the order of two consecutive types of moral judgment (wrongness vs. punishment). The aim was to examine the extent to which these two factors influence moral judgements.

There is a substantial body of literature that shows that moral judgements are influenced by factors that seem irrelevant because they are unrelated to the protagonists or the circumstances of their actions. People make different choices about identical situations depending on how these situations are formulated. For example, people are less harsh in their moral judgements when a situation is described as an act that would save 5 out of 10 people than when the same situation is described as an act that would let 5 out of 10 people die (this is known as the word framing effect first described by Tversky & Kahneman, 1981; see also Christensen & Gomila, 2012; Petrinovich & O’Neill, 1996). People also attribute less responsibility to an agent when the situation is described with abstract terms compared to emotional terms, supporting the idea that writing style influences the reader’s moral judgements (Christensen & Gomila, 2012; Schaich Borg, Hynes, Van Horn, Grafton, & Sinnott-Armstrong, 2006). The order in which information is presented also influences moral judgements. In the present paper, two types of order effects were examined: ‘within-scenario order effects’ which refer to the order in which the information was presented within the description of an event and ‘between-scenarios order effects’ which refer to the impact of the judgement of a previous event on the judgement of a subsequent event (Wiegmann, Okan, & Nagel, 2012).

### Within-scenario intention/outcome order effects

Cushman (2008) showed that healthy adults give more weight to the agent’s intention than the action outcome when they make moral judgements. Cushman suggested that this effect is explained by the intrinsic characteristic of intention information (see also Baez et al., 2014; Cushman, 2008; Moran et al., 2011; Young, Camprodon, Hauser, Pascual-Leone, & Saxe, 2010; Young, Cushman, Hauser, & Saxe, 2007; Young & Saxe, 2011). It remains so far unclear to what extent the weight given to intention can be affected by the order in which intention and outcome information is provided.

Piaget’s (1978) showed that young children base their moral judgements on the outcome of the action and that they take into account the agent’s intention only when they grow older. Since in Piaget’s (1978) study, the intention was always presented first and the outcome last, some authors have suggested that these results could at least in part be due to a recency effect, which young children would be more sensitive to (Austin, Ruble, & Trabasso, 1977; Enescu & Kuhn, 2012; Feldman, Klosson, Parsons, Rholes, & Ruble, 1976).

Recency effects were also found in adults’ moral judgements. However, this was shown in a study that did not specifically look at the order in which intention and outcome information was presented. Enescu & Kuhn (2012) asked judges to watch a mock trial in a courtroom displaying testimonies about a road traffic accident. The defendant was accused of assault through negligence and violation of duties. One testimony by a forensic expert was against the defense and the two other testimonies (one from a friend of the defendant and one from an independent eyewitness) were in favor of the defense. The order of the three testimonies was fully counterbalanced. Results showed significantly less condemnations when the independent eyewitness was placed in the last position.

The first aim of the present study was to clarify whether a recency effect could also be observed in adults’ moral judgements when manipulating the order of intention and outcome information.

### Between-scenarios punishment/wrongness order effects

Beside the within-scenario order effects mentioned above, several studies also showed that consecutive scenarios are not judged independently of each other. A moral principle activated for the evaluation of one particular scenario can be applied to the evaluation of a subsequent scenario, which is called the ‘transfer effect’ (Wiegmann et al., 2012). The transfer effect has been mainly studied using the classic trolley dilemmas (Horne, Powell, & Spino, 2013; Lanteri, Chelini, & Rizzello, 2008; Lombrozo, 2009; Schwitzgebel & Cushman, 2012; Wiegmann & Okan, 2012; Wiegmann et al., 2012; Wiegmann & Waldmann, 2014). In the trolley dilemmas, participants have to imagine a trolley going quickly on a railway where five men are working. If the participants do nothing, the trolley will kill these 5 workers. In the ‘Switch’ version of the dilemma, participants can activate a control stick which guides the trolley towards another railway where only one man works. In the ‘Push’ version of the dilemma, participants are on a bridge that steps over the railway with a big fat man stood on the bridge. Participants can push the big fat man on the railway to stop the trolley. Classically, while adults agree to redirect the trolley in the Switch version, they reject the idea to push the big fat man in the Push version. This response dissociation has been regularly shown despite both dilemmas being equivalent for the trade-off (five people saved for one sacrificed). It has been proposed that this response dissociation is due to the fact that these two types of dilemmas differ in terms of the intensity of the emotional response they generate, the Push version leading to a stronger emotional response (Greene, Sommerville, Nystrom, Darley, & Cohen, 2001). The difference in intensity of emotional response has been attributed to at least three characteristics of the Push version: the harm is inflicted by an action rather than by an omission, the action leads to a direct physical contact between the agent and the victim, and/or the sacrifice of the victim is used as a means rather than being collateral damage (see Cushman, Young et Hauser, 2006 for more information). Importantly, the transfer effect between these two dilemmas is asymmetrical. The evaluation of the Push dilemma affects the evaluation of the Switch dilemma, but the reverse is not true. Indeed, participants accept less the Switch dilemma after having judged the Push dilemma compared to when they judged the Switch dilemma alone or first. In contrast, the evaluation of the Switch dilemma first has no effect on the subsequent evaluation of the Push dilemma (Horne et al., 2013; Lanteri et al., 2008; Lombrozo, 2009; Schwitzgebel & Cushman, 2012; Wiegmann & Okan, 2012; Wiegmann et al., 2012; Wiegmann & Waldmann, 2014).

According to Wiegmann & Waldmann (2014), the asymmetrical transfer effect could be explained by the relative ambiguity of the dilemma and their default evaluation (i.e., the evaluation of the dilemma when presented alone). Regarding the relative ambiguity, the Push dilemma is unambiguous: there is no way to save the five men workers without killing the big fat man. In contrast, the Switch dilemma is relatively ambiguous. It could be that after being redirected, the trolley stops and does not kill anybody. Regarding the default evaluation, most people reject the action in the Push dilemma (negative default evaluation) while most people accept the action in the Switch dilemma (positive default evaluation). Wiegmann & Waldmann (2014) suggested that the evaluation of an ambiguous dilemma changes when it is immediately preceded by an unambiguous dilemma with a different default evaluation. For instance, a positive ambiguous dilemma (e.g., the Switch dilemma) would be evaluated less positively if it is preceded by a negative unambiguous dilemma (e.g., the Push dilemma). The reverse would not be true. The evaluation of an unambiguous dilemma would not depend on the presence of a previous dilemma irrespective of its ambiguity or its default evaluation valence. The second aim of the present study was to address this asymmetrical transfer effect for a type of moral judgement different from the one involved in the classic trolley dilemmas.

### The present study

Experiment 1 was aimed at clarifying the extent to which adults’ moral judgements can be impacted by the order in which information about the agent’s intention and action outcome is presented. If a recency effect applies to the processing of intention and outcome information, then the weight given to the intention should be stronger when the intention is present after the outcome, rather than before.

The aim of our second experiment was to address the existence of an asymmetrical transfer effect for moral judgements. There are several forms of moral judgements (wrongness, permissibility, blame and punishment judgements, see Cushman, 2008) and the same information is not necessarily used to make these different moral judgements. For example, Cushman (2008) showed that people rely both on outcome and intention during punishment judgements but rely more exclusively on intention during wrongness judgements. This would make punishment questions more ambiguous, especially when intention and outcome information do not lead to the same conclusion, as is the case in situations of accidental harm (no intention to harm, but harmful outcome) or attempted harm (intention to harm, but neutral outcome). In Experiment 2, we investigated whether the order in which participants were asked to think about whether an action is wrong or deserves punishment, affects their judgements in an asymmetrical way. The question of interest was whether this would cause a transfer effect between the questions asked (wrongness and punishment questions) and whether such a transfer would be asymmetrical, with the unambiguous situation (wrongness judgement) influencing a subsequent ambiguous situation (punishment judgement) but not the reverse. Because wrongness questions afford mainly to consider the agent’s intention, we hypothesized that answering wrongness questions first (e.g., How wrong was the agent’s behavior?) would result in participants giving more weight than usual to intention information when making subsequent punishment judgements (e.g., How much the agent’s behavior should be punished). This would be particularly true for accidental harm and attempted harm which are the most ambiguous scenarios.

To examine these order effects, and contrary to most previous studies, we used everyday life moral scenarios in which the actual or potential harm was not as severe as death (fall, food poisoning, etc.). Such scenarios are more representative of the kinds of moral judgement that people usually engage in (see the following link: https://doi.org/10.6084/m9.figshare.5117107.v1 for the full list of actual or potential harm used in the current study). In both experiments, intention (no intention to harm vs. intention to harm) and outcome information (neutral outcome vs. harmful outcome) were orthogonally manipulated leading to four conditions: neutral scenarios where the agent had no intention to harm and no harm occurred; accidental harm scenarios where the agent had no intention to harm but the harm occurred accidentally; attempted harm scenarios where the agent had the intention to harm but no harm occurred and; intentional harm scenarios where the agent had the intention to harm and the harm occurred. This allowed us to examine the relative weight given to intention and outcome information by contrasting the responses to these four types of scenarios.

## Experiment 1 – Within-Scenario Intention/Outcome Order Effects

This first experiment manipulated the order in which intention and outcome information was presented to adult participants (intention-outcome order vs. outcome-intention order) in order to examine whether the order had an impact on adult participants’ judgements.

### Material and methods

#### Participants

Forty healthy adult volunteers without any known psychiatric or neurological disorder participated in the study for course credits. They all had normal or corrected to normal vision, spoke French fluently and were between 18 and 25 years old. Written informed consent was obtained prior to the experiment. Participants did not report any expectations that were in line with our hypotheses during debriefing. The number of participants was decided a priori based on the number of participants usually tested for experiments in moral cognition using the same paradigm in our lab and in similar studies (Young et al., 2010, 2007; Young & Saxe, 2008, 2009). Data collection was completed before running analyses. Data from 3 participants were removed because of technical failures and alcohol consumption before the experiment. The data from the remaining 37 participants were analyzed (33 females, 27 right-handed, age range 18–25, *M* = 20.16; *SD* = 1.36). This study was approved by the Commission d’Éthique de l’Institut de Recherche en Sciences Psychologique de l’Université catholique de Louvain.

#### Design and materials

Participants judged the 64 verbal vignettes created by Leloup, Dongo Miletich, Andriet, Vandermeeren, & Samson (2016). The vignettes presented sequentially the context, the protagonist’s belief about the situation and his or her action (on the basis of which his or her intention to harm or not could be inferred) and the action outcome (i.e. whether it harmed or not another person).

The 64 vignettes presented familiar contexts to encourage everyday life moral judgements. They were always presented in the third-person perspective. The harm was always a physical injury which occurred by action (never by omission) in the absence of physical contact between the protagonists. The agent never benefitted from harming or not the victim. The agent and victim had the same gender, and they had names of equivalent frequency in the Belgian population. Their relationship was not defined in order to control the kinship/friendship effect.

Within vignettes, the intention to harm (no intention to harm vs. intention to harm) and the outcome of the action (neutral outcome vs. harmful outcome) were orthogonally manipulated leading to four conditions: neutral scenarios (i.e., no intention to harm – neutral outcome); accidental harm scenarios (i.e., no intention to harm – harmful outcome); attempted harm scenarios (i.e., intention to harm – neutral outcome) and; intentional harm scenarios (i.e., intention to harm – harmful outcome). In the present study, we additionally manipulated the order of presentation of intention and outcome information (intention followed by outcome vs. outcome followed by intention), which led to 8 experimental conditions. The 64 vignettes were adapted to the eight experimental conditions leading to a total of 512 stimuli (see Table 1 for examples).

**Table 1:** Examples of scenarios (English translation) used in Experiment 1.

Intention – Outcome order	Outcome – Intention order

***Neutral scenario:***	

Steve and Nathan restock the new merchandise on the shelves in the storehouse. Steve fills the top shelves while Nathan puts the products just below. Steve puts the box of tin cans on the top shelves. Steve thinks that the shelf **will not break** under the weight of the box. The shelf **does not break** and Nathan is **OK**.	Steve and Nathan restock the new merchandise on the shelves in the storehouse. Steve fills the top shelves while Nathan puts the products just below. Steve puts the box of tin cans on the top shelves. The shelf **does not break** and Nathan is **OK** Steve thought that the shelf **would not break** under the weight of the box.
***Accidental harm scenario:***

Steve and Nathan restock the new merchandise on the shelves in the storehouse. Steve fills the top shelves while Nathan puts the products just below. Steve puts the box of tin cans on the top shelves. Steve thinks that the shelf **will not break** under the weight of the box. The shelf **breaks** and Nathan is **hurt**.	Steve and Nathan restock the new merchandise on the shelves in the storehouse. Steve fills the top shelves while Nathan puts the products just below. Steve puts the box of tin cans on the top shelves. The shelf **breaks** and Nathan is **hurt**. Steve thought that the shelf **would not break** under the weight of the box.
***Attempted harm scenario:***

Steve and Nathan restock the new merchandise on the shelves in the storehouse. Steve fills the top shelves while Nathan puts the products just below. Steve puts the box of tin cans on the top shelves. Steve thinks that the shelf **will break** under the weight of the box. The shelf **does not break** and Nathan is **OK**.	Steve and Nathan restock the new merchandise on the shelves in the storehouse. Steve fills the top shelves while Nathan puts the products just below. Steve puts the box of tin cans on the top shelves. The shelf **does not break** and Nathan is **OK**. Steve thought that the shelf **would break** under the weight of the box.
***Intentional harm scenario:***

Steve and Nathan restock the new merchandise on the shelves in the storehouse. Steve fills the top shelves while Nathan puts the products just below. Steve puts the box of tin cans on the top shelves. Steve thinks that the shelf **will break** under the weight of the box. The shelf **breaks** and Nathan is **hurt**.	Steve and Nathan restock the new merchandise on the shelves in the storehouse. Steve fills the top shelves while Nathan puts the products just below. Steve puts the box of tin cans on the top shelves. The shelf **breaks** and Nathan is **hurt**. Steve thought that the shelf **would break** under the weight of the box.

Each participant judged each of the 64 vignettes once, eight vignettes per condition. A given vignette was judged by all the participants but in different experimental conditions (random distribution). The full set of vignettes is made freely available via the following link: https://doi.org/10.6084/m9.figshare.5117107.v1.

#### Procedure

**Figure 1:**
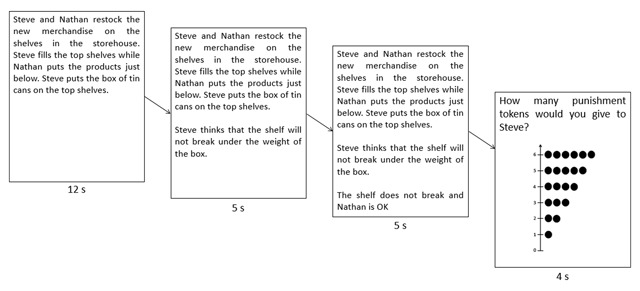
Illustration of the presentation of the vignettes in three cumulative segments, the question and the response scale given to the participants.

The 64 vignettes were presented in a pseudo-random order using Psychopy 1.79.00 (Peirce, 2007, 2009). The vignettes were presented in 3 cumulative segments (previous segments remained on the screen when later segments were added): (1) the contextual information and action (12s), (2) the protagonist’s intention or the action outcome (an additional 5s), (3) the action outcome or the protagonist’s intention (an additional 5s). This procedure allowed to control when the participant was exposed to each piece of information of the vignette (see Greene et al., 2001; Leloup et al., 2016). All of the story text was then removed from the screen and replaced by the question and a vertically presented response scale. Participants had 4s to judge ‘How many punishment tokens would you give to the agent?’ (Combien de jetons de punition donneriez-vous à l’agent?) on a 7-points response scale ranging from 0 ‘Not punished’ (Pas puni) to 6 ‘Strongly punished’ (Fortement puni; see Figure 1).

### Results

To test the effect of the order in which the intention and outcome information was presented, we conducted a repeated measures ANOVA on the ratings with Intention (no intention to harm vs. intention to harm), Outcome (neutral outcome vs. harmful outcome) and Order (intention-outcome vs. outcome-intention) as within-subject factors. We found a significant main effect of Intention (*F*_(1,36)_ = 244.76, *p* < .001, partial η^2^ = 0.872) with more severe ratings when there was an intention to harm (3.80 ± 0.17) compared to when there was no intention to harm (0.89 ± 0.10). There was also a significant main effect of Outcome (*F*_(1,36)_ = 135.748, *p* < .001, partial η^2^ = 0.79), with more severe ratings for harmful outcomes (2.92 ± 0.12) compared to neutral outcomes (1.77 ± 0.11).

These main effects were qualified by two significant two-way interactions. First, the Order by Intention interaction was significant (*F*_(1,36)_ = 4.213, *p* = .047, partial η^2^ = 0.105).^1^ Pairwise comparison with Bonferroni correction showed a significant effect of Order when there was an intention to harm, with more severe ratings when the harmful intention was presented last (*p* = .022, intention-outcome order: 3.74 ± 0.18, outcome-intention order: 3.86 ± 0.17). There was however, no significant effect of Order when there was no intention to harm (*p* = .315).

**Figure 2:**
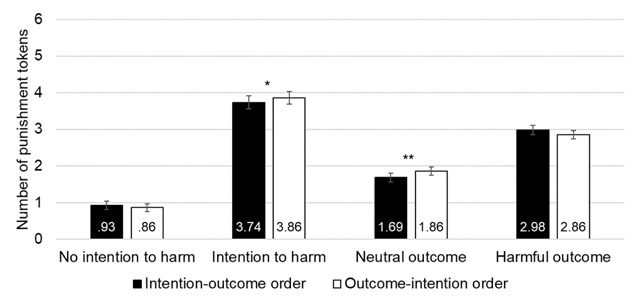
Results of Experiment 1. Mean number of punishment tokens attributed (0 ‘Not punished’ to 6 ‘Strongly punished’) as a function of the order in which the information was presented (intention-outcome order vs. outcome-intention order), the intention (no intention to harm vs. intention to harm) and the outcome (neutral outcome vs. harmful outcome). Error bars represent standard mean error. (*) = *p* < .10; * = *p* < .05; ** = *p* < .01.

The second significant interaction was between Order and Outcome (*F*_(1,36)_ = 13.054, *p* = .001, partial η^2^ = 0.266). Pairwise comparison with Bonferroni correction showed a significant effect of Order for neutral outcomes, with less severe punishment when the neutral outcome was presented last (*p* = .001, intention-outcome order: 1.69 ± 0.12; outcome-intention order: 1.86 ± 0.11), and no significant effect of order for harmful outcomes (*p* = .063; see Figure 2).^2^

**Figure 3:**
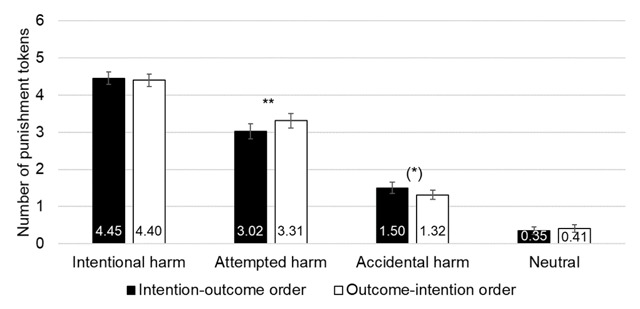
Results of Experiment 1. Mean number of punishment tokens attributed from 0 ‘Not punished’ to 6 ‘Strongly punished’ as a function of the order of information (intention-outcome order vs. outcome-intention order) and the scenario (intentional ham, attempted harm, accidental harm, neutral). Error bars represent standard mean error. (*) = *p* < 0.10; * = *p* < 0.05; ** = *p* < 0.01.

In order to test whether, as hypothesized, the order effect was shown for the scenarios where intention and outcome do not lead to the same conclusion (i.e., accidental and attempted harm), we tested the order effect for each scenario. Pairwise comparison with Bonferroni correction showed a significant effect of Order for attempted harm scenarios, with less severe punishment in the intention-outcome order (3.02 ± 0.21) than in the outcome-intention order (3.31 ± 0.20, Bonferroni: *p* < .001). Pairwise comparison with Bonferroni correction showed a marginally significant effect of Order for accidental harm scenarios, with more severe punishment in the intention-outcome order (1.50 ± 0.15) than in the outcome-intention order (1.32 ± 0.13, Bonferroni: *p* = .056).^3^ Thus, situations most prone to the order effect were situations in which intention and outcome conflicted with one another (see Figure 3).

**Figure 4:**
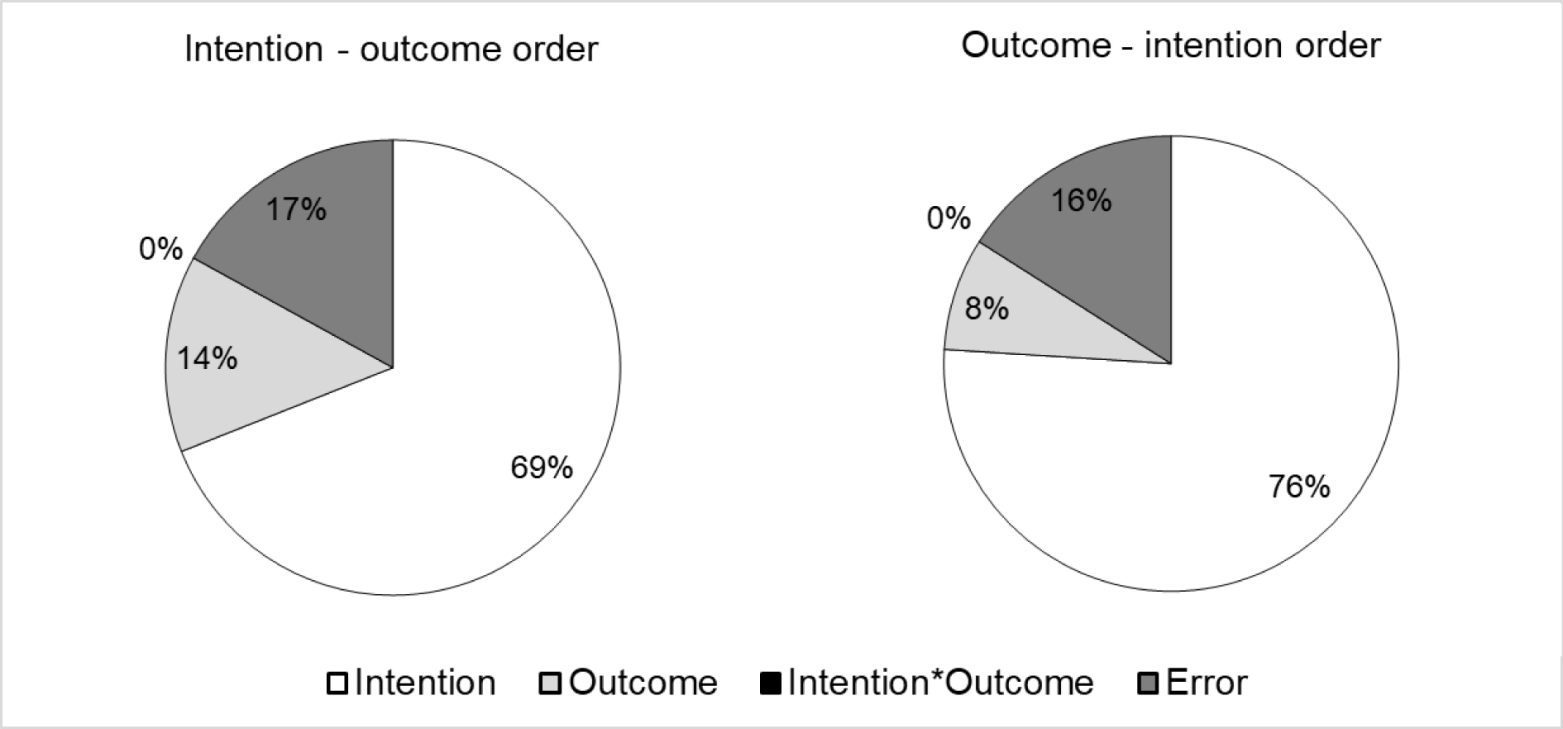
Results of Experiment 1. Proportion of within variability explained by each factor (intention, outcome, intention by outcome interaction and error) for the intention-outcome order (left) and for the outcome-intention order (right).

As in Cushman (2008), we explored how judgements of punishment differed in terms of the weight given to intention and outcome information, as a function of the order in which the information was provided (intention-outcome order vs. outcome-intention order). In order to do so, for each order condition we computed the sum of squares for each within-subject main effect (intention and outcome) and interaction (intention by outcome interaction) expressed as a proportion of the total variability (the total within sum of squares, including within sum of squared errors). The reliance on the outcome was higher in the intention-outcome order (14%) than in the outcome-intention order (8%). On the other hand, the reliance on the intention was less in the intention-outcome order (69%) than in the outcome-intention order (76%; see Figure 4). This is in line with the significant Order by Intention interaction (*F*_(1,36)_ = 4.213, *p* = .047, partial η^2^ = 0.105) and the significant Order by Outcome interaction (*F*_(1,36)_ = 13.054, *p* = .001, partial η^2^ = 0.266). The data can be accessed via the following link: https://doi.org/10.6084/m9.figshare.5117107.v1.

### Discussion

First of all, the parts of variance in the intention-outcome order (intention 69%, outcome 14%) were globally similar to those from Cushman (2008), which supports the validity of our francophone vignettes. With a punishment question and an intention-outcome order, Cushman (2008) had 68% of the variability for the intention factor^4^ and 20% of the variability for the outcome factor.

Secondly, participants made more severe punishment judgements when the intention to harm was described at the end of the scenario and they made less severe punishment judgements when the neutral outcome was described at the end of the scenario. Moreover, the analysis of variance showed that the variability explained by each factor (intention and outcome) was bigger when the factor was at the end of the scenario. When the outcome was presented at the end of the scenario, it explained 14% of the variability, but when it was presented at the beginning, it explained 8% of the variability. Similarly, when the intention was presented at the end of the scenario, it explained 76% of the variability compared to 69% when the intention was presented at the beginning. Our results are in line with the idea that the order of the information (intention-outcome order vs. outcome-intention order) modulates moral judgements and that such modulation takes the form of a recency effect. Such an intention/outcome order effect had however an impact on moral judgements only when the intention and outcome influenced judgements in opposite directions (i.e. in cases of attempted harm and to a lesser extent, accidental harm).

## Experiment 2 – Between-Scenarios Punishment/Wrongness Order Effects

The second experiment investigated the effect of the order in which adult participants were asked to think about whether the agent’s behavior was wrong (unambiguous judgement) or deserved punishment (ambiguous judgement). As explained before, the mechanisms activated for previous unambiguous judgements may influence subsequent ambiguous judgements. We hypothesized that responding first to wrongness questions (e.g., How wrong was the agent’s behavior?’ – A quel point est-ce mal de se comporter comme l’agent?) would impact on subsequent punishment judgements (e.g., How much the agent’s behavior should be punished?’ – A quel point est-ce punissable de se comporter comme l’agent?). This would be particularly true for accidental harm and attempted harm which correspond to the most ambiguous scenarios. We further hypothesized that the reverse order effect would not be observed. In order to test these hypotheses, we manipulated the order of the questions.

### Materials and methods

#### Participants

Sixty-eight healthy adult volunteers without any known psychiatric or neurological disorder participated in this study for credit course. The number of participants was decided a priori based on the number of participants usually tested for experiments in moral cognition using the same paradigm in our lab and in similar studies (Young et al., 2010, 2007; Young & Saxe, 2008, 2009). They all had normal or corrected to normal vision, spoke French fluently and were between 18 and 25 years old. Written informed consent was obtained prior to the experiment. Data from six participants were removed because of technical or human errors. All 62 remaining participants (53 female, 55 right-handed, age range 18–25 *M* = 20.34; *SD* = 1.23) were assigned following a random procedure to one of the two experimental conditions defined by the order of the question (30 participants in the wrongness-punishment question group and 32 participants in the punishment-wrongness question group). The two groups were equivalent in terms of age (*t*_(60)_ = 0.792, *p* = .431) and gender (χ^2^(1) = 1.409; *p* = .294). During post-experiment briefing, participants did not report any expectations that were in line with our hypotheses. The study was approved by the Commission d’Éthique de l’Institut de Recherche en Sciences Psychologique de l’Université catholique de Louvain.

#### Design and Material

Participants were presented with the same 64 vignettes as those used in Experiment 1, except that we did not manipulate the order in which intention and outcome information was presented (intention was always presented first) in order not to make the design unnecessarily complex. The intention to harm (no intention to harm vs. intention to harm) and the action outcome (neutral outcome vs. harmful outcome) were manipulated as within-subject factors. The 64 vignettes were adapted to four experimental conditions (neutral, accidental harm, attempted harm and intentional harm scenarios). The order of the questions varied across participants: 30 participants had to answer the wrongness question first followed by the punishment question (wrongness-punishment group) and 32 participants had to answer the punishment question first followed by the wrongness question (punishment-wrongness group).

Each participant evaluated each of the 64 vignettes once. The 64 vignettes were split in two blocks of 32 vignettes. The two blocks were counterbalanced in terms of the question and the order, so it was not always the same vignettes that were judged with the wrongness or the punishment question, and it was not always the same vignettes that were judged in the first block or the second block. Each block included 8 vignettes by condition (neutral, accidental harm, attempted harm, intentional harm scenarios, see Table 2 for examples). These conditions were also counterbalanced so that a given vignette was judged once by all the participants, but in different experimental conditions (random distribution). The full set of vignettes is made freely available via the following link: https://doi.org/10.6084/m9.figshare.5117107.v1.

**Table 2:** Examples of scenarios (English translation) used in Experiment 2.

	No intention to harm	Intention to harm

**Neutral Outcome**	***Neutral scenario:*** Steve and Nathan restock the new merchandise on the shelves in the storehouse. Steve fills the top shelves while Nathan puts the products just below. Steve puts the box of tin cans on the top shelves. Steve thinks that the shelf **will not break** under the weight of the box. The shelf **does not break** and Nathan is **OK**.	***Attempted harm scenario:*** Steve and Nathan restock the new merchandise on the shelves in the storehouse. Steve fills the top shelves while Nathan puts the products just below. Steve puts the box of tin cans on the top shelves. Steve thinks that the shelf **will break** under the weight of the box. The shelf **does not break** and Nathan is **OK**.
**Harmful Outcome**	***Accidental harm scenario:*** Steve and Nathan restock the new merchandise on the shelves in the storehouse. Steve fills the top shelves while Nathan puts the products just below. Steve puts the box of tin cans on the top shelves. Steve thinks that the shelf **will not break** under the weight of the box. The shelf **breaks** and Nathan is **hurt**.	***Intentional harm scenario:*** Steve and Nathan restock the new merchandise on the shelves in the storehouse. Steve fills the top shelves while Nathan puts the products just below. Steve puts the box of tin cans on the top shelves. Steve thinks that the shelf **will break** under the weight of the box. The shelf **breaks** and Nathan is **hurt.**

#### Experimental Design and procedure

The vignettes were presented in a pseudo-random order using Psychopy 1.79.(Peirce, 2007, 2009). We used the same format of cumulative presentation of segments as in Experiment 1. Subjects had 4s to judge ‘How much the agent’s behavior should be punished?’ (A quel point est-ce punissable de se comporter comme l’agent?) or ‘How wrong was the agent’s behavior?’ (A quel point est-ce mal de se comporter comme l’agent?) on the 7-points response scale ranging from 0 ‘Not at all’ (Pas du tout) to 6 ‘Very much’ (Tout à fait). Participants were randomly assigned to one of the two experimental conditions that defined the order of question presentation (wrongness-punishment group and punishment-wrongness group). For the participants from the wrongness-punishment order, the first part of the experiment (1 block of 32 vignettes, 8 vignettes by condition) was presented with a wrongness question. The second part (1 block of 32 vignettes, 8 vignettes by condition) was then presented with a punishment question. The participants from the punishment-wrongness order had the counterbalanced presentation.

### Results

To examine the effect of the order of question presentation, we conducted a mixed ANOVA on the ratings with Question (wrongness vs. punishment), Intention (no intention to harm vs. intention to harm) and Outcome (neutral outcome vs. harmful outcome) as within-subject factors and with Order (wrongness-punishment vs. punishment-wrongness) as a between-subjects factor. We found a significant main effect of Question (*F*_(1,60)_ = 10.382, *p* = .002, partial η^2^ = 0.148), with more severe ratings for the wrongness question (2.93 ± 0.08) compared to the punishment question (2.69 ± 0.09). There was also a significant main effect of Intention (*F*_(1,60)_ = 504.584, *p* < .001, partial η^2^ = 0.894), with more severe ratings for intention to harm (4.23 ± 0.11) compared to no intention to harm (1.39 ± 0.08). We also found a significant main effect of Outcome (*F*_(1,60)_ = 205.558, *p* < .001, partial η^2^ = 0.774), with more severe ratings for harmful outcomes (3.36 ± 0.08) compared to neutral outcomes (2.26 ± 0.09).

**Figure 5:**
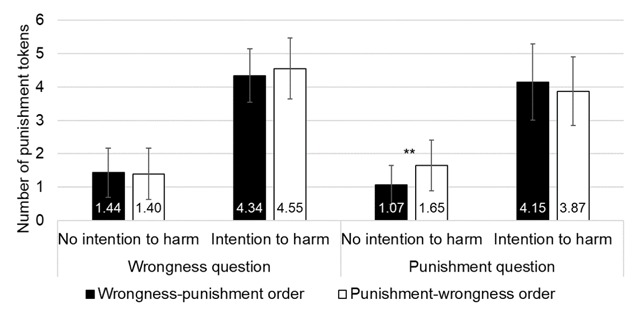
Results of Experiment 2. Mean ratings attributed from 0 ‘Not at all’ to 6 ‘Very much’ as a function of the order of the question (wrongness-punishment vs. punishment-wrongness), the question (wrongness vs. punishment) and the intention (no intention to harm vs. intention to harm). Error bars represent standard mean error. (*) = *p* < .10; * = *p* < .05; ** = *p* < .01.

The Question by Intention interaction was significant (*F*_(1,60)_ = 7.796, *p* = .007, partial η^2^ = 0.115) and qualified by a significant Question by Intention by Order interaction (*F*_(1,60)_ = 16.656, *p* < .001, partial η^2^ = 0.217). To decompose this triple interaction, we conducted a mixed ANOVA with Intention (no intention to harm vs. intention to harm) as a within-subject factor and Order (wrongness-punishment vs. punishment-wrongness) as a between-subjects factor separately for each type of question. For the wrongness question, the results showed a significant main effect of Intention (*F*_(1,60)_ = 472.959, *p* < 0.001, partial η^2^ = 0.887), no significant main effect of Order (*F*_(1,60)_ = 0.317, *p* = .575, η^2^ = 0.005) and no significant interaction effect (*F*_(1,60)_ = 0.76, *p* = .387, partial η^2^ = 0.013). For the punishment question, the results showed a significant main effect of Intention (*F*_(1,60)_ = 323.258, *p* < .001, partial η^2^ = 0.843), no significant main effect of Order (*F*_(1,60)_ = 0.748, *p* = .391, η^2^ = 0.012) but a significant Intention by Order interaction (*F*_(1,60)_ = 8.554, *p* = .005, partial η^2^ = 0.125). The decomposition of the Intention by Order interaction showed that when there was no intention to harm, participants gave significantly less severe punishment ratings when the punishment question was presented after the wrongness question compared to when the punishment question was presented first (wrongness-punishment order: 1.07 ± 0.13, punishment-wrongness order: 1.65 ± 0.12; Bonferroni: *p* = .001). In contrast, there was no significant effect of Order when there was an intention to harm (Bonferroni: *p* = .314; see Figure 5).

**Figure 6:**
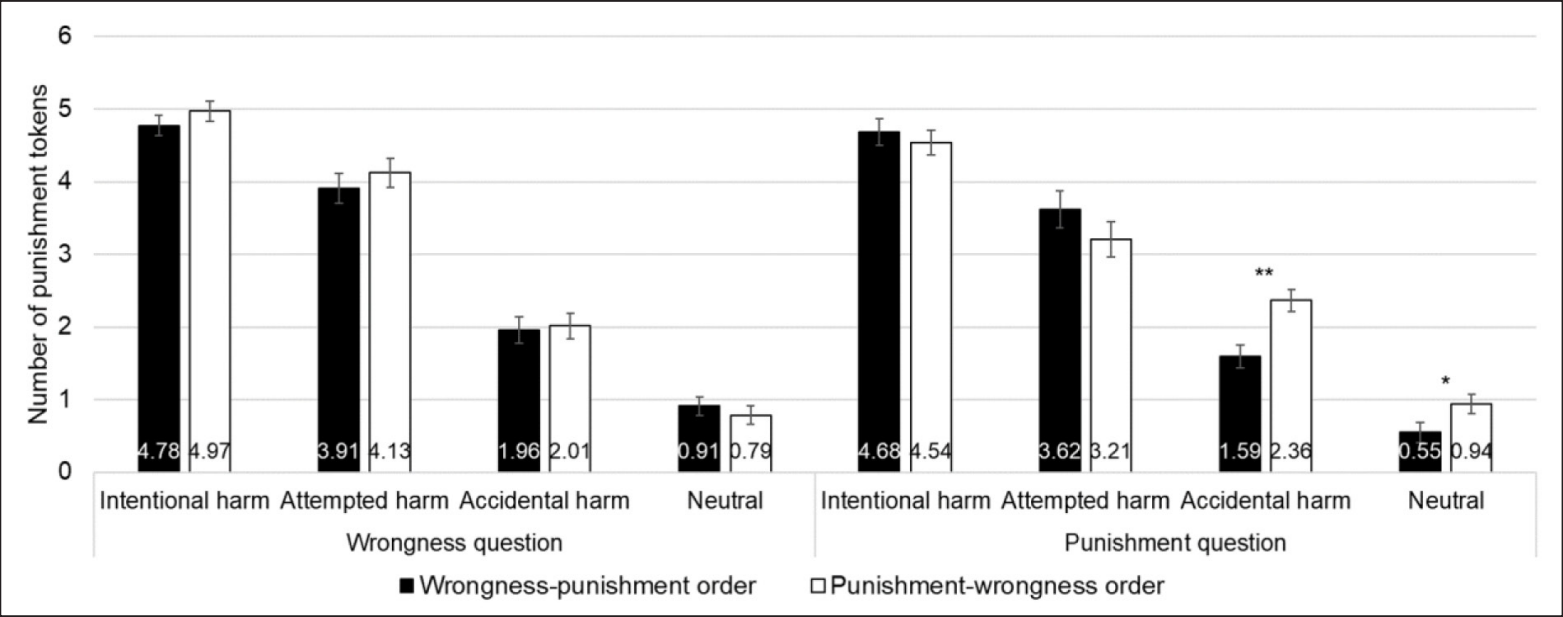
Results of Experiment 2. Mean ratings attributed from 0 ‘Not at all’ to 6 ‘Very much’ as a function of the question order (wrongness-punishment order vs. punishment-wrongness order), the question (wrongness question vs. punishment question) and the scenario (intentional ham, attempted harm, accidental harm, neutral). Error bars represent standard mean error. (*) = *p* < .10; * = *p* < .05; ** = *p* < .01.

In order to further explore the data, we analyzed which scenario was impacted by the order of the questions for the punishment question. Pairwise comparison with Bonferroni correction showed a significant effect of Order for accidental harm scenarios, with less severe punishment in the wrongness-punishment order (1.59 ± 0.16) than in the punishment-wrongness order (2.36 ± 0.16, Bonferroni: *p* = .001). Pairwise comparison with Bonferroni correction showed a significant effect of Order for neutral scenarios, with less severe punishment in the wrongness-punishment order (0.55 ± 0.14) than in the punishment-wrongness order (0.94 ± 0.13, Bonferroni: *p* = .041). For the wrongness question, none of the pairwise comparisons with Bonferroni correction showed a significant effect (see Figure 6).

**Figure 7:**
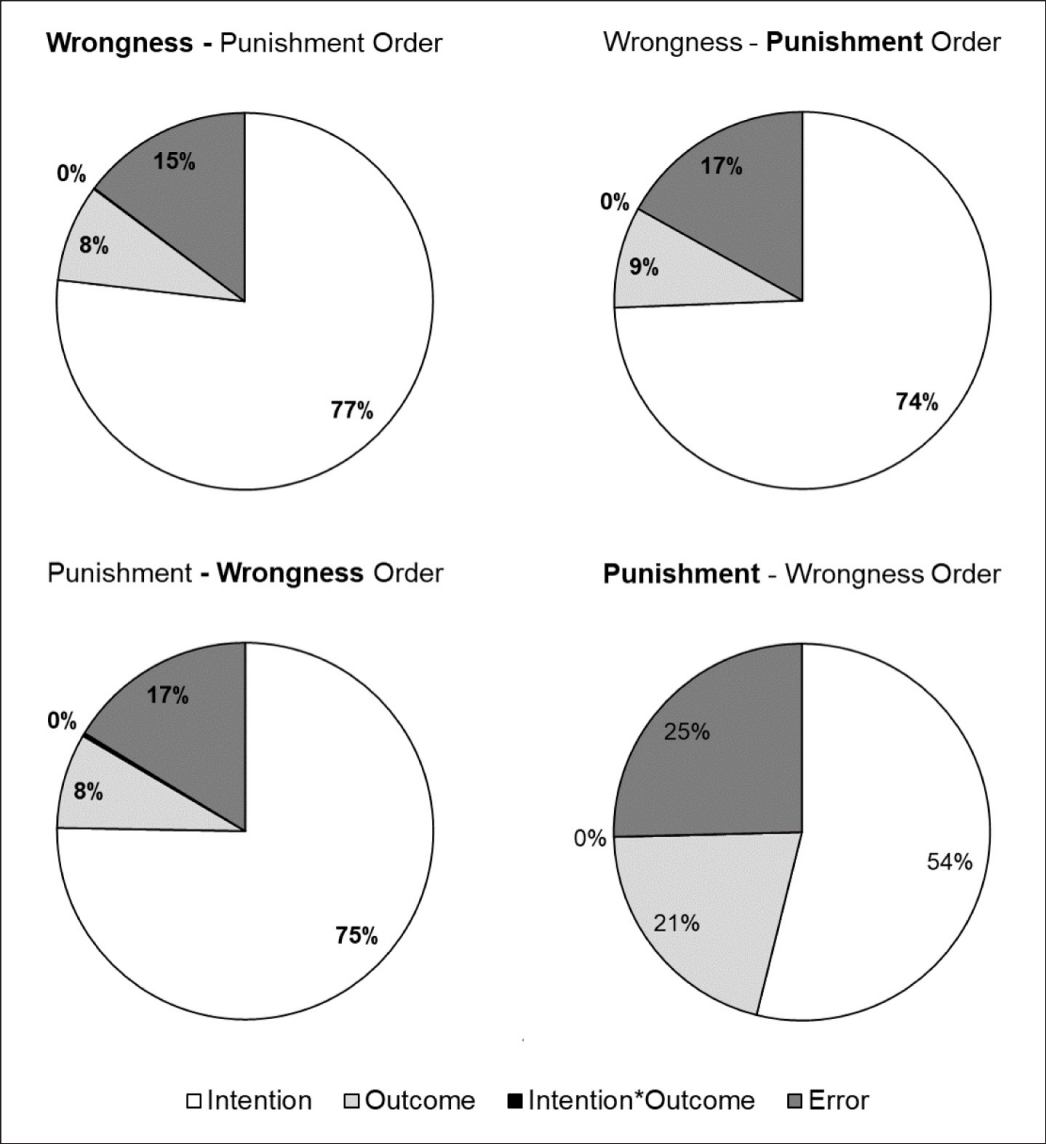
Results of Experiment 2. Proportion of within variability explained by each factor (intention, outcome, intention by outcome interaction and error) for the wrongness question when it was asked in the wrongness-punishment order (upper panel left) and in the punishment-wrongness order (lower panel left), for the punishment question when it is asked in the wrongness-punishment order (upper panel right) and in the punishment-wrongness order (lower panel right).

We then explored whether judgements of wrongness and punishment differed in their reliance on intention and outcome information depending on the question and the order of the question. For the Wrongness question, the reliance on intention and outcome was globally similar when the wrongness question was asked first (Wrongness first: Intention = 77% of the variability; Outcome = 8% of the variability) compared to when it was asked last (Wrongness second: Intention = 75% of the variability; Outcome = 8% of the variability). The absence of interaction effect (*F*_(1,60)_ = 0.76, *p* = .387, partial η^2^ = 0.013) mentioned above for the wrongness question was confirmed by the absence of an order effect for this question. However, for the Punishment question, the reliance differed when the punishment question was asked first (Punishment first: Intention = 54% of the variability; Outcome = 21% of the variability) compared to when it was asked last (Punishment second: Intention = 74% of the variability; Outcome = 9% of the variability), with more reliance on intention when the punishment question was asked last (see Figure 7). This is in line with the significant Intention by Order interaction (*F*_(1,60)_ = 8.554, *p* = .005, partial η^2^ = 0.125) for the punishment question mentioned above. The data can be accessed via the following link: https://doi.org/10.6084/m9.figshare.5117107.v1.

### Discussion

First of all, the parts of variance were again similar to those reported by Cushman (2008). Cushman reported 83% of the variability by the intention factor and 3% of variability by the outcome factor for the wrongness question, while these number were respectively 77% and 8% in the present experiment. Cushman reported 68% (intention) and 20% (outcome) for the punishment question, while in the present experiment, analysis showed respectively 54% and 21%. These similarities confirm again the validity of our francophone vignettes.

Secondly, as a reminder, because punishment questions are more ambiguous than wrongness questions, we hypothesized that responding first to wrongness questions would impact on subsequent punishment judgements, but that the reverse would not be observed. This asymmetrical order effect would be particularly true for accidental harm and attempted harm scenarios, which are the most ambiguous. Results were in line with this expected asymmetrical order effect. When participants responded to punishment questions after having responded to wrongness questions, they relied more on intention, and judged scenarios with no intention to harm (neutral and accidental harm scenarios) as significantly less punishable compared to when they answered the punishment questions first. However, answering punishment questions first had no significant impact on the subsequent answers to wrongness questions. This result is in line with our hypothesis that only ambiguous judgements (i.e. in our experiment punishment judgements) would be influenced by the mechanisms activated during the prior unambiguous judgements (wrongness judgements). We expected the effect to be particularly marked for scenarios where intention and outcome lead to conflicting judgements. The transfer effect was indeed significant for accidental harm but it was not significant for attempted harm (even though the numbers were in the expected direction). The transfer effect also extended to neutral scenarios. We will come back to this effect in the general discussion.

## General Discussion

Across two experiments we showed that both within- and between-scenarios order effects significantly impacted the extent to which people considered the agent’s intention and the action outcome during their everyday life moral judgements. More particularly, we showed that when manipulating the order in which intention and outcome information is provided within a vignette, a recency effect can be observed. Furthermore, when manipulating between-scenarios, the order of the requested moral judgements (wrongness-punishment vs. punishment-wrongness) led to a transfer from the less ambiguous (wrongness) to the most ambiguous question (punishment), but not vice versa. These effects are discussed in turn.

### Within-scenario intention/outcome order effects

The first experiment investigated if the order of the information (intention followed by outcome vs. outcome followed by intention) could impact on moral judgements and whether such impact would take the form of a recency effect as suggested by some studies (Austin et al., 1977; Enescu & Kuhn, 2012; Feldman et al., 1976). Results showed that participants gave more punishment when the intention to harm was at the end of the scenario, and gave less punishment when the neutral outcome was at the end of the scenario. Moreover, the analysis of variance showed that the variability explained by each factor (intention and outcome) was bigger when the factor was at the end of the scenario. Our results are thus in line with the idea that the order of the information inside the scenario modulates moral judgements and that this takes the form of a recency effect. This recency effect is compatible with the recency effects observed in the literature investigating the effect of the order in which intention and outcome information on children’s moral judgements (Austin et al., 1977; Feldman et al., 1976). Our findings also extend the finding by Enescu & Kuhn (2012) who showed a recency effect in relation to the order of presentation of testimonies in a mock trial.

Even though a clear recency effect was found in our experiment, overall, participants still relied more on intention than outcome irrespective of the order in which the information was presented. This effect, which is compatible with Cushman’s (2008) original results, suggests that participants rely more on intention than outcome because of an intrinsic characteristic of the intention information. There are thus separate and co-existing mechanisms driving the importance given to intention information and the recency effect. It is noteworthy that intention could be easily inferred and was thus largely unambiguous in both Cushman’s (2008) study and the present study. Yet, in some daily life situations, someone else’s intention can be more ambiguous. It would be worth investigating the extent to which people still rely heavily on intention during moral judgements when intention must be inferred based on the agent’s external actions.

Finally, we expected the order effect to be particularly noticeable when there was a conflict between intention and outcome, namely for accidental harm and attempted harm. This is what we observed, although the order effect on accidental harm was marginal and the order effect was much larger on attempted harm. Indeed, judgements of attempted harm were more severe when the harmful intention was presented last, while judgements of accidental harm were only slightly more lenient when the absence of intention to harm was presented last. This could be due to the fact that attempted harm is subject to a combined effect of intention and negative information causing greater salience above any order effect (Ito, Larsen, Smith, & Cacioppo, 1998; Smith, Cacioppo, Larsen, & Chartrand, 2003). Indeed, negative information is more salient than neutral information due to its emotional nature (Ito, Larsen, Smith, & Cacioppo, 1998; Smith, Cacioppo, Larsen, & Chartrand, 2003).

### Between-scenarios punishment/wrongness order effects

The second experiment investigated the effects of the order in which people were asked to judge whether the agent’s behavior was wrong or deserved punishment. Previous between-scenarios order manipulations in the classic trolley dilemmas showed asymmetrical transfer effects. Ambiguous judgements were influenced by prior unambiguous judgements, while unambiguous judgements were not influenced by prior judgements (Wiegmann & Waldmann, 2014). Given that during punishment judgements, both intention and outcome information are taken into account, while during wrongness judgements intention information is taken into account more exclusively (Cushman, 2008), we assumed that punishment judgements were more ambiguous than wrongness judgements. Accordingly, if the asymmetrical transfer effect observed in the trolley dilemmas generalizes to other types of moral judgements, we expected to find an influence of wrongness judgements on subsequent punishment judgements, but not the reverse. This is exactly what we observed. Thus, the wrongness judgement, which unambiguously made participants focus on intention, modulated subsequent punishment judgement by framing participants to focus more on intention than they would normally do.

We also expected that the transfer effect would be most marked for the most ambiguous scenarios, i.e. the accidental and attempted harm scenarios in which there is a conflict between intention and outcome. The transfer effect was not significant for attempted harm (although there was a trend in the expected direction) but it was highly significant for accidental harm. Several studies in the literature have highlighted that the conflict between intention and outcome is larger for accidental than attempted harm (Buon, Seara-Cardoso, & Viding, 2016; Cushman, Sheketoff, Wharton, & Carey, 2013). It has been proposed that in accidental harm scenarios, the processing of the harmful outcome triggers a negative emotional response which gives more weight to the analysis of the outcome and its causes. Such analysis competes with the processing of the mitigating circumstances associated with the absence of intention to harm. In the case of attempted harm scenarios, the absence of a harmful outcome does not trigger such fine-grained outcome analysis and moral judgement is made more easily on the basis of intention (Buon et al., 2016; Cushman et al., 2013). The stronger transfer effect for accidental harm can thus be explained by this stronger conflict.

Unexpectedly, the transfer effect was also significant for neutral scenarios where there is in principle no conflict between intention and outcome. Provided this effect is not a false positive (the effect was indeed much smaller), two explanations could be put forward. One possible explanation is that the transfer effect could have framed participants to focus more on intention than they would normally do, not to the detriment of the outcome analysis, but to the detriment of other variables such as the severity of the potential harm caused. Indeed, if there were no other variables than intention and outcome to be taken into account, we would expect neutral scenarios to be judged as deserving no punishment at all, i.e. a score of 0. Yet the actual mean score was 0.94 (punishment – wrongness order) and 0.55 (wrongness – punishment order) indicating that participants may have taken into account other factors (e.g., the negligence of the agent in the face of the potential harm caused). Another possible explanation could be linked to Rosset’s (2008) observation that there is an intentionality bias in ambiguous situations, i.e. an implicit bias whereby all actions are judged to be intentional by default. The neutral intention scenarios are the sole scenarios where the intentionality bias has to be overridden. The transfer effect could have allowed participants to better override the intentionality bias and so to fully take into account the neutral intention.

### Conclusion

An important goal of moral cognition research is to understand how people make moral judgements. Our results contribute to highlighting the importance of considering apparently irrelevant factors related to information presentation. These factors can impact, not only on the conclusions that researchers make based on their experimental design (Bartels, Bauman, Cushman, Pizarro, & McGraw, 2015), but more importantly conclusions that people make in real life situations. We would like to think that human adults base their everyday life moral judgements on relevant information only, but we have to keep in mind that moral judgements can be impacted by morally irrelevant factors unrelated to the protagonists or the circumstances of the action such as order effects. Such effects seem to be particularly noticeable when judgements are difficult to make, that is, exactly in those situations were decisions cannot be entirely rule-based but require human judgement.

## Additional File

The additional file for this article can be found as follows:

- pb-58-1-421-s1.pdf
  **Appendix** Bayesian analyses carried out by using JASP 0.8.0.1. DOI: https://doi.org/10.5334/pb.421.s1

## Footnotes

See “Appendix” for the corresponding Bayesian analyses carried out by using JASP 0.8.0.1 (JASP Team, 2016).

See “Appendix” for the corresponding Bayesian analyses carried out by using JASP 0.8.0.1 (JASP Team, 2016).

See “Appendix” for the corresponding Bayesian analyses carried out by using JASP 0.8.0.1 (JASP Team, 2016).

In his study, Cushman (2008) manipulated desire (desire to harm vs. no desire to harm), belief (believe the action will harm vs. believe the action will not harm) and outcome (neutral outcome vs. harmful outcome) factors. So we compared the combined parts of variance of his desire and belief factors to the parts of variance obtained for our intention factor.

## References

[1] Austin, V. D., Ruble, D. N., & Trabasso, T. (1977). Recall and Order Effects as Factors in Children’s Moral Judgments. Child Development, 48(2), 470–474. Retrieved from: http://www.jstor.org/stable/1128641 DOI: 10.2307/1128641

[2] Baez, S., Couto, B., Torralva, T., Sposato, L. A., Huepe, D., Montanes, P., Ibanez, A., et al. (2014). Comparing Moral Judgments of Patients With Frontotemporal Dementia and Frontal Stroke. JAMA Neurology, 71(9), 1172–1176. DOI: 10.1001/jamaneurol.2014.347 25047907

[3] Bartels, D. M., Bauman, C. W., Cushman, F. A., Pizarro, D. A., & McGraw, A. P. (2015). Moral Judgment and Decision Making In: Keren, G., & Wu, G. (eds.), The Wiley Blackwell Handbook of Judgment and Decision Making, 1–51. Wiley: Chichester, UK DOI: 10.1002/9781118468333.ch17

[4] Buon, M., Seara-Cardoso, A., & Viding, E. (2016). Why (and how) should we study the interplay between emotional arousal, Theory of Mind, and inhibitory control to understand moral cognition? Psychonomic Bulletin & Review, 23(6), 1660–1680. DOI: 10.3758/s13423-016-1042-5 27169411 PMC5133272

[5] Christensen, J. F., & Gomila, A. (2012). Moral dilemmas in cognitive neuroscience of moral decision-making: A principled review. Neuroscience and Biobehavioral Reviews, 36(4), 1249–1264. DOI: 10.1016/j.neubiorev.2012.02.008 22353427

[6] Cushman, F. (2008). Crime and punishment: Distinguishing the roles of causal and intentional analyses in moral judgment. Cognition, 108(2), 353–80. DOI: 10.1016/j.cognition.2008.03.006 18439575

[7] Cushman, F., Sheketoff, R., Wharton, S., & Carey, S. (2013). The development of intent-based moral judgment. Cognition, 127(1), 6–21. DOI: 10.1016/j.cognition.2012.11.008 23318350

[8] Cushman, F., Young, L., & Hauser, M. (2006). The role of conscious reasoning and intuition in moral judgment: Testing the three principles of harm. Psychological Science, 17(12), 1082–1089. DOI: 10.1111/j.1467-9280.2006.01834.x 17201791

[9] Enescu, R., & Kuhn, A. (2012). Serial effects of evidence on legal decision-making. The European Jounral of Psychology Applied to Legal Context, 4(2), 99–118.

[10] Feldman, N. S., Klosson, E. C., Parsons, J. E., Rholes, W. S., & Ruble, D. N. (1976). Order of Information Presentation and Children’s Moral Judgments. Child Development, 47, 556–559. DOI: 10.2307/1128821 1269325

[11] Greene, J. D., Sommerville, R. B., Nystrom, L. E., Darley, J. M., & Cohen, J. D. (2001). An fMRI investigation of emotional engagement in moral judgment. Science (New York, N.Y.), 293(5537), 2105–8. DOI: 10.1126/science.1062872 11557895

[12] Horne, Z., Powell, D., & Spino, J. (2013). Belief Updating in Moral Dilemmas. Review of Philosophy and Psychology, 4, 705–714. DOI: 10.1007/s13164-013-0159-y

[13] Ito, T. A., Larsen, J. T., Smith, N. K., & Cacioppo, J. T. (1998). Negative information weighs more heavily on the brain: The negativity bias in evaluative categorizations. Journal of Personality and Social Psychology, 75(4), 887–900. DOI: 10.1037/0022-3514.75.4.887 9825526

[14] JASP Team. (2018). JASP (Version 0.9) downloaded free of charge from jasp-stats.org.

[15] Lanteri, A., Chelini, C., & Rizzello, S. (2008). An Experimental Investigation of Emotions and Reasoning in the Trolley Problem. Journal of Business Ethics, 83, 789–804. DOI: 10.1007/s10551-008-9665-8

[16] Leloup, L., Dongo Miletich, D., Andriet, G., Vandermeeren, Y., & Samson, D. (2016). Cathodal Transcranial Direct Current Stimulation on the Right Temporo-Parietal Junction Modulates the Use of Mitigating Circumstances during Moral Judgments. Frontiers in Human Neuroscience, 10, 355 7 DOI: 10.3389/fnhum.2016.00355 27462213 PMC4940443

[17] Lombrozo, T. (2009). The Role of Moral Commitments in Moral Judgment. Cognitive Science, 33, 273–286. DOI: 10.1111/j.1551-6709.2009.01013.x 21585471

[18] Moran, J. M., Young, L., Saxe, R., Lee, S. M., O’Young, D., Mavros, P. L., & Gabrieli, J. D. (2011). Impaired theory of mind for moral judgment in high-functioning autism. Proceedings of the National Academy of Sciences of the United States of America, 108(7), 2688–92. DOI: 10.1073/pnas.1011734108 21282628 PMC3041087

[19] Peirce, J. W. (2007). PsychoPy-Psychophysics software in Python. Journal of Neuroscience Methods, 162(1–2), 8–13. DOI: 10.1016/j.jneumeth.2006.11.017 17254636 PMC2018741

[20] Peirce, J. W. (2009). Generating Stimuli for Neuroscience Using PsychoPy. Frontiers in Neuroinformatics, 2, 10 1 DOI: 10.3389/neuro.11.010.2008 19198666 PMC2636899

[21] Petrinovich, L., & O’Neill, P. (1996). Influence of Wording and Framing Effects on Moral Intuitions. Ethology and Sociobiology, 17, 145–171. DOI: 10.1016/0162-3095(96)00041-6

[22] Piaget, J. (1978). La contrainte adulte et le réalisme moral. In: Le jugement moral chez l’enfant, 81–155. Retrieved from: http://www.fondationjeanpiaget.ch/fjp/site/crypt/verifier.php?DOCID=1594.

[23] Rosset, E. (2008). It’s no accident: Our bias for intentional explanations. Cognition, 108, 771–780. DOI: 10.1016/j.cognition.2008.07.001 18692779

[24] Schaich Borg, J., Hynes, C., Van Horn, J., Grafton, S., & Sinnott-Armstrong, W. (2006). Consequences, Action, and Intention as Factors in Moral Judgments: An fMRI Investigation. Journal of Cognitive Neuroscience, 18(5), 803–817. DOI: 10.1162/jocn.2006.18.5.803 16768379

[25] Schwitzgebel, E., & Cushman, F. (2012). Expertise in Moral Reasoning? Order Effects on Moral Judgment in Professional Philosophers and Non-Philosophers. Mind & Language, 27(2), 135–153. DOI: 10.1111/j.1468-0017.2012.01438.x

[26] Smith, N. K., Cacioppo, J. T., Larsen, J. T., & Chartrand, T. L. (2003). May I have your attention, please: Electrocortical responses to positive and negative stimuli. Neuropsychologia, 41(2), 171–183. DOI: 10.1016/S0028-3932(02)00147-1 12459215

[27] Tversky, A., & Kahneman, D. (1981). The Framing of Decisions and the Psychology of Choice. Science, 211(4481), 453–458. DOI: 10.1126/science.7455683 7455683

[28] Wiegmann, A., & Okan, Y. (2012). Order ffects in Moral Judgment Searching for an Explanation. In: Proceedings of the Thirty-Fourth Annual Conference of the Cognitive Science Society Sapporo, Japan.

[29] Wiegmann, A., Okan, Y., & Nagel, J. (2012). Order Effects in Moral Judgments. Philosophical Psychology, 25(6), 813–836. DOI: 10.1080/09515089.2011.631995

[30] Wiegmann, A., & Waldmann, M. R. (2014). Transfer effects between moral dilemmas: A causal model theory. Cognition, 131(1), 28–43. DOI: 10.1016/j.cognition.2013.12.004 24440432

[31] Young, L., Camprodon, J. A., Hauser, M., Pascual-Leone, A., & Saxe, R. (2010). Disruption of the right temporoparietal junction with transcranial magnetic stimulation reduces the role of beliefs in moral judgments. Proceedings of the National Academy of Sciences of the United States of America, 107(15), 6753–8. DOI: 10.1073/pnas.0914826107 20351278 PMC2872442

[32] Young, L., Cushman, F., Hauser, M. D., & Saxe, R. (2007). The neural basis of the interaction between theory of mind and moral judgment. Proceedings of the National Academy of Sciences of the United States of America, 104(20), 8235–40. DOI: 10.1073/pnas.0701408104 17485679 PMC1895935

[33] Young, L., & Saxe, R. (2008). The neural basis of belief encoding and integration in moral judgment. NeuroImage, 40(4), 1912–20. DOI: 10.1016/j.neuroimage.2008.01.057 18342544

[34] Young, L., & Saxe, R. (2009). Innocent intentions: A correlation between forgiveness for accidental harm and neural activity. Neuropsychologia, 47(10), 2065–72. DOI: 10.1016/j.neuropsychologia.2009.03.020 19467357

[35] Young, L., & Saxe, R. (2011). When ignorance is no excuse: Different roles for intent across moral domains. Cognition, 120(2), 202–14. DOI: 10.1016/j.cognition.2011.04.005 21601839

