# Supplementary material for: Moral Judgments Depend on Information Presentation: Evidence for Recency and Transfer Effects
Source: Psychol Belg. 2018 Sep 27;58(1):256–75. doi: 10.5334/pb.421 (PMC6194526; doi:10.5334/pb.421)
Supplement: Supplementary file 1 [file pb-58-1-421-s1.pdf]

Appendix

[1]Bayesian analysis for the Order by Intention interaction (F(1,36) = 4.213, p = .047, partial η² = 0.105) observed in Experiment 1 – Within-Scenario Intention/Outcome Order Effects.

Bayesian analyses were carried out using JASP 0.8.0.1 (JASP Team, 2016) in order to quantify the extent to which data support a model with the Order by intention interaction against any model without this interaction. We included the Order, the Intention and the Outcome as nuisance variables, meaning that these variables were included in all models (including the null model). The Bayes factor (BF10) represents the degree to which the data are more likely under the model with the Order by Intention interaction compared to the null model. Results showed that the data was 40.30 times more likely under the model with the interaction, BF10 = 2.564e+13 (see Table 3), supporting a presence of this interaction effect. In other words, data were more likely under a model that did include the Order by Intention interaction.

[Insert Table 3 about here]

Table 3: Bayesian Repeated-measure ANOVA table for the Order by Intention interaction (F(1,36) = 4.213, p = .047, partial η² = 0.105) observed in Experiment 1

**Model Comparison**	**P(M)**	**P(M|data)**	**BF_M**	**BF_{10}**	**Error %**
Null model	0.111	3.161e -30	2.529e -29	1.000	
**Order ✻ Intention**	0.111	8.107e -17	6.486e -16	**2.564e +13**	6.616
Order ✻ Outcome	0.111	1.287e -18	1.030e -17	4.071e +11	6.516
Order ✻ Intention + Order ✻ Outcome	0.111	0.666	15.938	2.106e +29	10.186
Intention ✻ Outcome	0.111	8.614e -31	6.891e -30	0.272	5.975
Order ✻ Intention + Intention ✻ Outcome	0.111	2.595e -17	2.076e -16	8.209e +12	10.056
Order ✻ Outcome + Intention ✻ Outcome	0.111	3.943e -19	3.154e -18	1.247e +11	8.289
Order ✻ Intention + Order ✻ Outcome + Intention ✻ Outcome	0.111	0.279	3.100	8.833e +28	20.098
Order ✻ Intention + Order ✻ Outcome + Intention ✻ Outcome + Order ✻ Intention ✻ Outcome	0.111	0.055	0.465	1.738e +28	7.266

Note. All models include Order, Intention, Ouctome, subject.

[2]**Bayesian Analysis for the paired-samples T-Test performed between both order (intention-outcome order vs. outcome-intention order) for the harmful outcomes (<u>p=.063</u>) in Experiment 1 – Within-Scenario Intention/Outcome Order Effects.**

Bayes Factors (BF) were calculated for the paired-samples T-Test between the two order conditions (intention-outcome vs. outcome-intention) for the harmful outcomes. Bayes Factors, calculated using JASP software, revealed that the Bayes Factor, for the comparison between intention-outcome order and outcome-intention order for the harmful outcomes is BF_{10} = 0.919 (see table 4). This analysis suggests that there is no evidence for a difference between intention-outcome order and outcome-intention order for the harmful outcomes (Jeffreys, 1961 cited by Wagenmakers et al., 2016).

[Insert Table 4 about here]

Table 4: Bayesian Paired Samples T-Test table performed between both order (intention-outcome order vs. outcome-intention order) for the harmful outcomes

**Harmful outcome**			**BF_{10}**	**error %**
**<u>Intention-Outcome order</u>**	-	Outcome-Intention order	**<u>0.919</u>**	1.355e -7

[3]**Bayesian Analysis for the paired-samples T-Test between both order (intention-outcome order vs. outcome-intention order) for accidental harm (Bonferroni : p = .056) and attempted harm (Bonferroni : p < .001) in Experiment 1 – Within-Scenario Intention/Outcome Order Effects.**

Bayes Factors (BF) were calculated for the paired-samples T-Test between the two order conditions (intention-outcome order vs. outcome-intention order) for accidental harm and attempted harm. Bayes Factors, calculated using JASP software, revealed that the Bayes Factor, for the comparison between intention-outcome order and outcome-intention order for the accidental harm is BF_{10} = 1.013 (see table 5). This analysis suggests that there is no evidence for a difference between intention-outcome order and outcome-intention order for accidental harm (Jeffreys, 1961 cited by Wagenmakers et al., 2016).
Bayes Factors, calculated using JASP software, revealed that the Bayes Factor, for the comparison between intention-outcome order and outcome-intention order for the attempted harm is BF_{10} = 144.552 (see table 5). This analysis suggests that there is strong evidence for a difference between intention-outcome order and outcome-intention order for attempted harm (Jeffreys, 1961 cited by Wagenmakers et al., 2016).

[Insert Table 5 about here]

Table 5: Bayesian Paired Samples T-Test table between both order (intention-outcome order vs. outcome-intention order) for accidental harm and attempted harm

		BF_{10}	error %
**Accidental harm**	- Order effect	**1.013**	1.654e -7
**Attempted harm**	- Order effect	**144.552**	2.692e -8
